# Supplementary material for: The Relationship between Narrative Skills and Executive Functions across Childhood: A Systematic Review and Meta-Analysis
Source: Children (Basel). 2023 Aug 15;10(8):1391. doi: 10.3390/children10081391 (PMC10453360; doi:10.3390/children10081391)
Supplement: Supplementary file 1 [file children-10-01391-s001.zip › Supplementary Material 1_ Additional figures and analyses/children-2533410-supplementary.pdf]

Supplementary Materials

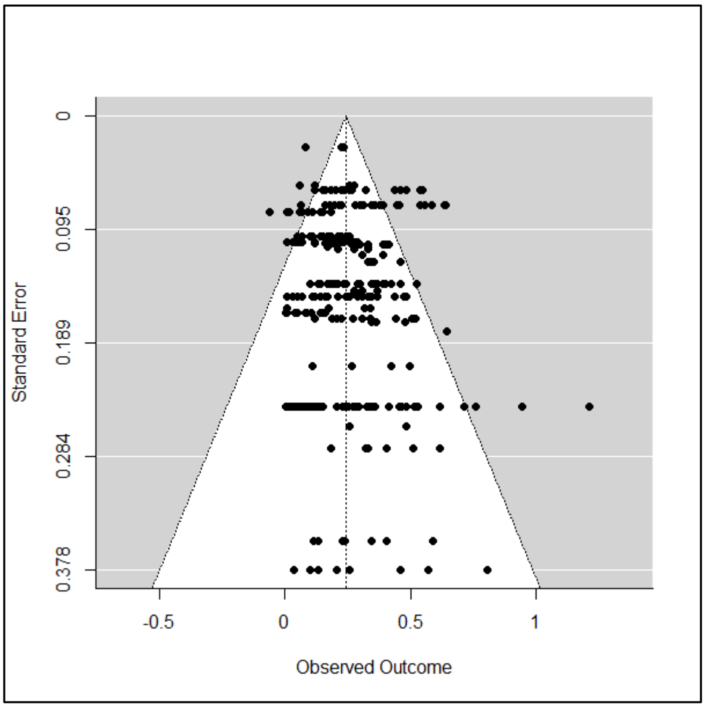

Figure S1. Funnel plot of the meta-analysis of main outcomes of all studies. Each plotted point represents the standard error and the *z* coefficient of the association between NC and EF, for a single outcome. The white triangle represents the region where 95% of the data points are expected to lie in the absence of publication bias. The vertical line represents the estimated effect size, based on the meta-analysis.

Investigation of potential moderators in the overall sample of studies

Table S1 reports the results of moderation analyses in the overall sample of studies included (n = 30). For categorical moderators, we report the coefficients and tests for the moderation (which indicated the difference between the two or more categories), and for the intercepts based on each level of the variable (dummy coded, indicating the effect size for each category separately). We categorized the sample in typically developing and atypically developing participants based on the presence of a diagnose (i.e., deafness, language impairment, neurodevelopmental disorders such as learning disorders or autism spectrum disorder). The analysis indicated that the effect size did not differ between the groups, $F(1, 285) = 0.010$, $p = .919$.

Looking at characteristics of the competences examined, we analyzed if written versus oral narrative tasks and micro versus macrostructural levels of narrative moderate the overall effect size of the relation between EF and NC. Results indicated that the effect size did not differ on type of narratives (i.e., written versus oral form), $F(1, 285) = 1.855$, $p = .174$. However, the magnitude of the effect size seem to vary depending on the NC competence considered, $F(1, 277) = 5.110$, $p = .024$.

The effect size was statistically higher for macrostructural (z = .269) than microstructural narrative competence (z = .223). As far as EF domains are concerned, results indicated that the overall effect size did not depend on type of EF process taken into consideration (i.e., interference control, inhibitory control, working memory capacity, updating of working memory, shifting, planning), $F(5, 261) = 0.842$, $p = .520$.

Table S1 Moderation analyses in the overall sample of studies included.

Effect	No. outcomes	No. studies	No. participants	Estimated z	SE	95% CI		p-value
Population								
Typically developing	203	21	3250	*0.243*	*0.039*	*-0.081*	*0.0736*	*0.919*
Atypically developing	84	9	346	*0.247*	*0.036*	*<.001*	*0.174*	*0.319*
EF domains								
Working memory capacity	79	19	2893	*0.266*	*0.037*	*-0.023*	*0.123*	*.182*
Working memory updating	14	2	77	*0.217*	*0.081*	*-0.158*	*0.161*	*.987*
Interference control	62	7	1544	*0.290*	*0.047*	*-0.019*	*0.166*	*.121*
Behavioral Inhibition	34	10	640	*0.216*	*0.034*	*0.148*	*0.284*	*<.001*
Shifting	50	11	736	*0.247*	*0.037*	*-0.042*	*0.104*	*.401*
Planning	28	7	485	*0.217*	*0.042*	*-0.081*	*0.084*	*.970*
Narrative Form								
Oral	171	6	266	*0.269*	*0.026*	*0.217*	*0.321*	*<.001*
Written	116	11	2535	*0.219*	*0.037*	*-0.123*	*0.022*	*.174*
Narrative Competence								
Micro-structural	150	20	3054	*0.223*	*0.020*	*-0.087*	*-0.006*	*0.024*
Macro-structural	129	23	1921	*0.269*	*0.021*	*0.226*	*0.312*	*<.001*

Note. Italic text indicates the levels of the categorical variables.
