# Supplementary material for: The Relationship between Narrative Skills and Executive Functions across Childhood: A Systematic Review and Meta-Analysis
Source: Children (Basel). 2023 Aug 15;10(8):1391. doi: 10.3390/children10081391 (PMC10453360; doi:10.3390/children10081391)
Supplement: Supplementary file 1 [file children-10-01391-s001.zip › Supplementary Material 2_ R code/R code syntax.pdf]

based on

Harrer, M., Cuijpers, P., Furukawa, T. A., & Ebert, D. D. (2021). *Doing meta-analysis with R: a hands-on guide*. Chapman and Hall/CRC.

We used the syntax for the purposes of the present meta-analysis, and we make it available for transparency of our analyses.

```
#open metafor library

library(metafor)

#compute main meta-analysis

full.model <- rma.mv (yi = Fisher_Z,
V = VarianzaFisher_Z,
slab = Study_ID,
data = meta_data,
random = ~ 1 | Study_ID/ID_Effect,
test = "t",
method = "REML")
summary (full.model)


#convert z to r

library (esc)
convert_z2r(0.24)

#compute variance of the 3-level model

i2 <- var.comp(full.model)
summary(i2)

#compute 2-level model

i3.removed <- rma.mv(yi = Fisher_Z,
V = VarianzaFisher_Z,
slab = Study_ID,
data = meta_data,
random = ~ 1 | Study_ID/ID_Effect,
test = "t",
method = "REML",
sigma2 = c(0, NA))
summary(i3.removed)
```

```
#compare multilevel vs non-multilevel model

anova(full.model, i3.removed)

# moderation analysis on the full sample of studies included:
# moderators in order: age in years/ age in age-bands (preschool; school-age; adolescence)/Literacy acquisition (3-7 years vs 8-15 year)/ type of narration form (oral vs written) / type of narrative competence (micro vs macrostructural level) / type EF processes (working memory capacity; updating; interefernce control; inhibitory control; shifting, planning) / Developmental status (typical vs atypical development)

mod.model.AgeYear <- rma.mv(yi = Fisher_Z,
V = VarianzaFisher_Z,
slab = Study_ID,
data = meta_data,
random = ~ 1 | Study_ID/ID_Effect,
test = "t",
method = "REML",
mods = ~ AgeYear)
summary(mod.model.AgeYear)

mod.model.AgeBand <- rma.mv(yi = Fisher_Z,
V = VarianzaFisher_Z,
slab = Study_ID,
data = meta_data,
random = ~ 1 | Study_ID/ID_Effect,
test = "t",
method = "REML",
mods = ~ AgeBand)
summary(mod.model.AgeBand)

mod.model.Literacy <- rma.mv(yi = Fisher_Z,
V = VarianzaFisher_Z,
slab = Study_ID,
data = meta_data,
random = ~ 1 | Study_ID/ID_Effect,
test = "t",
method = "REML",
mods = ~ Literacy)
summary(mod.model.Literacy)


mod.model.NarrativeType <- rma.mv(yi = Fisher_Z,
V = VarianzaFisher_Z,
slab = Study_ID,
```

```
data = meta_data,
random = ~ 1 | Study_ID/ID_Effect,
test = "t",
method = "REML",
mods = ~ Narrative_Type)
summary(mod.model.NarrativeType)

mod.model.Narrative_Comp <- rma.mv(yi = Fisher_Z,
V = VarianzaFisher_Z,
slab = Study_ID,
data = meta_data,
random = ~ 1 | Study_ID/ID_Effect,
test = "t",
method = "REML",
mods = ~ Narrative_Comp)
summary(mod.model.Narrative_Comp)

mod.model.EF_Comp <- rma.mv(yi = Fisher_Z,
V = VarianzaFisher_Z,
slab = Study_ID,
data = meta_data,
random = ~ 1 | Study_ID/ID_Effect,
test = "t",
method = "REML",
mods = ~ EF_Comp)
summary(mod.model.EF_Comp)

mod.model.Develop <- rma.mv(yi = Fisher_Z,
V = VarianzaFisher_Z,
slab = Study_ID,
data = meta_data,
random = ~ 1 | Study_ID/ID_Effect,
test = "t",
method = "REML",
mods = ~ Develop)
summary(mod.model.Develop)


#SEPARATE META-ANALYSIS PRE-LITERACY (13 studies) E POST-LITERACY ACQUISITION (16 studies).
1 study has been excluded because it did not report precise information on mean age of
participants.

# Oral vs Written (only done on post literacy data subsample)
mod.model.NarrativeType <- rma.mv(yi = Fisher_Z,
V = VarianzaFisher_Z,
```

```
slab = Study_ID,
data = data_postliteracy,
random = ~ 1 | Study_ID/ID_Effect,
test = "t",
method = "REML",
mods = ~ Narrative_Type)
summary(mod.model.NarrativeType)


# EF PROCESSES: Working memory capacity; Updating; Shifting; Inhibitory Control; Interference Control; Planning
#(pre and post literacy, respectively: note “pre” and “post” word in the object)

mod.pre.model.EF_Comp <- rma.mv(yi = Fisher_Z,
V = VarianzaFisher_Z,
slab = Study_ID,
data = data_preliteracy,
random = ~ 1 | Study_ID/ID_Effect,
test = "t",
method = "REML",
mods = ~ EF_Comp)
summary(mod.pre.model.EF_Comp)

mod.post.model.EF_Comp <- rma.mv(yi = Fisher_Z,
V = VarianzaFisher_Z,
slab = Study_ID,
data = data_postliteracy,
random = ~ 1 | Study_ID/ID_Effect,
test = "t",
method = "REML",
mods = ~ EF_Comp)
summary(mod.post.model.EF_Comp)

# NARRATIVE COMPETENCE: MICRO VS MACRO (pre and post literacy respectively)

mod.pre.model.Narrative_Comp <- rma.mv(yi = Fisher_Z,
V = VarianzaFisher_Z,
slab = Study_ID,
data = data_preliteracy,
random = ~ 1 | Study_ID/ID_Effect,
test = "t",
method = "REML",
mods = ~ Narrative_Comp)
summary(mod.pre,model.Narrative_Comp)
```

```
mod.post.model.Narrative_Comp <- rma.mv(yi = Fisher_Z,
V = VarianzaFisher_Z,
slab = Study_ID,
data = data_postliteracy,
random = ~ 1 | Study_ID/ID_Effect,
test = "t",
method = "REML",
mods = ~ Narrative_Comp)
summary(mod.post,model.Narrative_Comp)

# DEVELOPMENTAL STATUS: TYPICAL VS ATYPICAL DEVELOPMENT (pre and post literacy
respectively)

mod.pre.model.Develop <- rma.mv(yi = Fisher_Z,
V = VarianzaFisher_Z,
slab = Study_ID,
data = data_preliteracy,
random = ~ 1 | Study_ID/ID_Effect,
test = "t",
method = "REML",
mods = ~ Develop)
summary(mod.pre.model.Develop)

mod.post.model.Develop <- rma.mv(yi = Fisher_Z,
V = VarianzaFisher_Z,
slab = Study_ID,
data = data_postliteracy,
random = ~ 1 | Study_ID/ID_Effect,
test = "t",
method = "REML",
mods = ~ Develop)
summary(mod.post.model.Develop)

#Performing bias and sensitivity diagnostics

funnel(full.model)

#Colored funnel plot

cols <- palette.colors(length(unique(meta_data$Study_ID)), palette="Alphabet")
cols <- cols[as.numeric(factor(meta_data$Study_ID))]
funnel(full.model, col=cols)

#Performing Egg's test, based on https://stats.stackexchange.com/questions/155693/metafor-package-bias-and-sensitivity-diagnostics
```

```
Egg <- rma.mv (yi = Fisher_Z,
V = VarianzaFisher_Z,
slab = Study_ID,
data = meta_data,
random = ~ 1 | Study_ID/ID_Effect,
test = "t",
method = "REML",
mods = ~ SE_FisherZ)
summary (Egg)
```

```
#show standardized residuals and the leverages along the diagonal or the entire hat matrix to discover influential studies.

rstandard(full.model)

hatvalues(full.model)

cooks.distance(full.model)
```
