# Supplementary material for: The Relationship between Narrative Skills and Executive Functions across Childhood: A Systematic Review and Meta-Analysis
Source: Children (Basel). 2023 Aug 15;10(8):1391. doi: 10.3390/children10081391 (PMC10453360; doi:10.3390/children10081391)
Supplement: Supplementary file 1 [file children-10-01391-s001.zip › Supplementary Material 3_ Dataset and codebook/Codebook.pdf]

Meta Data

The file data 'meta_data.xlxs' contains all relevant information and the computed Fisher's *z* and its standard error, together with all relevant moderators.
Please note that the comma (,) is used as a separator for the decimals.

Variables included

ID_Effect	Univocal identifier of each effect size (1-287)
Article	Identifier of each article (1-30)
Study_ID	Univocal identifier of each study (1-25)
Authors	Identifier of the authors of each study.
Title_Art	Title of each article
Location	Country in which the study has been carried out.
YearPub	Year of publication of each study (1996-2021). One couple of authors has more than one study published in the same year (i.e., Swanson & Berninger). In this case, for the last publication, letter "b" follows the publication year (e.g., 1996b).
TypeP	Published or unpublished study (e.g., doctoral thesis)
Develop	Typical vs Atypical development of the participants in each study
Atyp	Diagnose of the atypically-developing participants
N sample	Number of participants in each study
AgeMonth	Mean age of participants expressed in months
AgeYear	Mean age of participants expressed in years
Literacy	This variable serves to indicate two different time windows.<br>Pre = studies with participants aged 3-7 years old; Post = studies with participants aged 8-18 years old
EF_Comp	Type of EF domains coded (WM capacity; WM updating; Behavioural Inhibition; Interference Control; Shifting; Planning).
Narrative_Type	Oral vs written form of narrative tasks administered in each study.
Narrative_Comp	Microstructural vs macrostructural competence analyzed in each study
R_Effect	Pearson product-moment correlation of the relation between EF and NC
Fisher_Z	Fisher's z coefficient, computed using Olkin and Finn's formula (2008).
F_CI_down	Lower bound of 95% confidence interval of the Fisher's *z*
F_CI_up	Upper bound of the 95% confidence interval of the Fisher's *z*
VarianceFisher_Z	Variance of the Fisher's *z*
SE_FisherZ	Standard error of the Fisher's *z*
